# Limited Clinical Efficacy with Potential Adverse Events in a Pilot Study of Autologous Adoptive Cell Therapy in Canine Oral Malignant Melanoma

**DOI:** 10.3390/vetsci11040150

**Published:** 2024-03-28

**Authors:** Yuan-Yuan Xia, Kwan-Hwa Chi, Albert Taiching Liao, Jih-Jong Lee

**Affiliations:** 1 Department and Graduate Institute of Veterinary Medicine, School of Veterinary Medicine, National Taiwan University, Taipe 10617, Taiwan; f05629021@ntu.edu.tw (Y.-Y.X.); atliao@ntu.edu.tw (A.T.L.); 2 National Taiwan University Veterinary Hospital, College of Bioresources and Agriculture, National Taiwan University, Taipei 10672, Taiwan; 3 Graduate Institute of Veterinary Clinical Science, School of Veterinary Medicine, National Taiwan University, Taipei 10617, Taiwan; m006565@ms.skh.org.tw; 4 Department of Radiation Therapy & Oncology, Shin Kong Wu Ho-Su Memorial Hospital, Taipei 11101, Taiwan

**Keywords:** dog, oral malignant melanoma, immunotherapy, adoptive cell therapy, natural killer cell

## Abstract

**Simple Summary:**

The pilot study explored a type of adoptive cell therapy in canine oral malignant melanoma, by collecting peripheral blood mononuclear cells from tumor-bearing dogs, expanding the cells to a predominantly non-B non-T population in vitro, and re-infusing the cells into dogs. Ten dogs were enrolled. The treatment was well tolerated in seven dogs, while the other three had suspected treatment-related anaphylaxis. The treatment efficacy was limited to a 49-day median progression-free interval, and dogs with progressive disease during treatment had shorter survival. Most of the recruited patients were in a later clinical stage and had macroscopic disease, which might affect the treatment efficacy. Further exploration of this kind of cell therapy in an adjuvant setting could be considered with adequate protocol modification and standardization.

**Abstract:**

Adoptive cell therapy (ACT) has been studied in several human and canine cancers with some promising clinical outcomes but not in canine oral malignant melanoma (OMM). Our manuscript aimed to explore one kind of ACT, the ex vivo-expanded autologous immune cell infusion in canine OMM, as this tumor remains a treatment dilemma. The study recruited dogs with histopathological diagnoses of oral malignant melanoma, generated their peripheral blood mononuclear cells, expanded them into predominantly non-B non-T cells via stimulations of IL-15, IL-2, and IL-21, and then re-infused the cells into tumor-bearing dogs. Ten dogs were enrolled; three dogs did not report any adverse events; three had a mildly altered appetite; one had a mildly increased liver index, while the other three developed suspected anaphylaxis at different levels. The median progression-free interval was 49 days. Dogs with progressive disease during treatment had a shorter survival. This pilot study indicates limited efficacy with potential adverse events of this ACT. Most recruited patients were in a later stage and had macroscopic disease, which might affect the treatment efficacy. Further exploration of this cell therapy in an adjuvant setting, with adequate protocol modification and standardization, could still be considered.

## 1. Introduction

Canine oral malignant melanoma (OMM) presents considerable treatment challenges due to local invasiveness and common distant metastasis. Surgery and radiation therapy (RT) provide the most effective locoregional tumor control; however, systemic treatments such as chemotherapy and targeted therapy have only limited efficacy [1]. Melanoma is regarded as an immunogenic tumor in both humans and dogs, leading to the potential of immunotherapy as a treatment option to reverse the lost equivalence between the immune system and tumor cells. Currently, the only approved canine melanoma immunotherapy is the DNA vaccine Oncept^TM^, while some ongoing studies of immune checkpoint blockers such as anti-PD-1 [2,3] and anti-PD-L1 [4] antibodies also showed promising results, and the commercialized anti-PD-L1 antibody Gilvetmab was conditionally USDA-approved for malignant melanoma treatment in late 2023. In addition, applying an adjuvant anti-CSPG4 DNA vaccine in combination with radical surgery also revealed benefits in canine OMM management [5].

Another kind of immunotherapy, adoptive cell therapy (ACT), or adoptive cell transfer, involves the ex vivo generation and expansion of lymphocytes and the subsequent re-infusion of those lymphocytes into cancer patients to achieve therapeutic effects in both human and veterinary cancer fields [6]. Different kinds of lymphocytes can be employed for ACT, including tumor-infiltrating lymphocytes (TILs) obtained from tumor tissue, expanded and activated peripheral blood-derived lymphocytes, or lymphocytes engineered with chimeric antigen receptors (CARs) [7]. CAR T-cell therapy has been approved by the FDA for human hematopoietic cancers and has also been reported in canine lymphoma [8,9]. The adverse events, especially the cytokine-release syndrome and neurological side effects, as well as the expense, are some crucial limitations. As early as the 1980s, Dr. Rosenberg’s team reported clinical applications of lymphokine-activated killer cells (LAKs) generated from peripheral blood, as well as TILs obtained from autologous tumor tissue, in human cancer treatment. Although the sources differed, the LAKs and TILs were activated similarly by utilizing interleukin (IL)-2 and human serum. The objective tumor responses were 44% (LAKs) and 40–60% (TILs) in conjunction with IL-2 [10,11]. TIL infusion treatment was found to be more potent in mediating tumor regression in murine models [10], but was seldom reported in veterinary medicine.

Peripheral blood-derived LAKs were obtained by IL-2 stimulation. Similarly, peripheral blood mononuclear cells (PBMCs) can also be activated via other lymphokine or cytokine cocktails, as reported in the veterinary oncology research field. T lymphocytes or T killer cells from either healthy [12] or tumor-bearing dogs [13] can be successfully expanded and activated ex vivo, with the aid of anti-CD3 antibody, IL-2, with or without other cytokines such as interferon α (IFN-α). The autologous lymphokine-activated T killer cells were reinfused into tumor-bearing dogs, and a subsequent increase in CD8+ T cells was observed, without any adverse events after repeated infusions [13]. Under different culturing conditions, O’Connor et al. [14] reported that non-specific T cells could be expanded via artificial antigen-presenting cells and lymphokine combinations of IL-2 and IL-21, and the autologous cell transfer significantly extended the progression-free interval in dogs with Non-Hodgkin B-cell lymphoma who had undergone regular CHOP chemotherapy. Another team [15] reported a retrospective study that propagated T cells using a similar method. The study combined CHOP chemotherapy, autologous peripheral blood hematopoietic stem cell transplants, and ACT as multimodal treatments in dogs with high-grade B-cell lymphoma. Although no significant survival difference was reached, the treatment was safe in all patients. In addition, in canine appendicular osteosarcoma, a clinical trial involving an autologous cancer cell vaccine, adoptive T-cell transfusion, and IL-2 was accomplished in 14 dogs [16]. The result was promising, with a median survival time of 415 days and a 36% 2-year survival rate. The toxicity was minimal with appropriate pre-medications. According to the studies mentioned above, although the manufacturing was different, at least the T-cell ACT was deemed safe, with or without clinical efficacy.

Except for T cells, the natural killer (NK) cells play a vital role in bridging innate and adaptive immunity, generating an anti-tumor response in a “missing-self” recognition process [17] and exhibiting tumor-killing effects. Tumor cells may down-regulate their major histocompatibility complex (MHC) expression to evade recognition and elimination by T cells, whereas NK cells can attack those tumor cells with abnormal MHC expression, making them a potent candidate for ACT with a lower risk of graft-versus-host adverse events [17,18]. In human research, killer-cell ACT involves two different types, the LAK and the cytokine-induced killer (CIK) cells, and the CIK cells involve a mixed phenotype of T cells and natural killer cells (e.g., CD3+CD56+) [19]. Both types have shown clinical benefits in several types of human cancers, including hepatocellular carcinoma, lung cancer, colorectal cancer, renal cell carcinoma, ovarian cancer, and multiple myeloma [19,20]. In the veterinary oncology field, Canter et al. assessed the effect of radiation combined with local injection of IL-12 and autologous NK cells in both osteosarcoma mouse models and dog patients [21,22]. NK cell cytotoxicity and homing ability were found to be enhanced after radiation in cell line and mouse model experiments. The multitherapy in 10 dogs with locally advanced osteosarcoma resulted in a 50% six-month metastasis-free rate, despite some cases that had local infection and systemic grade 3 toxicity [21]. This marked the first clinical trial in dogs using NK cells in this modality. Additionally, there was a case report of two aged cats who received LAK therapy after diagnosis of squamous carcinoma and mammary gland adenocarcinoma, showing no significant toxicity or changes in quality of life after repeated infusions [23]. Briefly, studies about killer-cell ACT were fewer than T-cell ACT in the veterinary field.

Although research on ACT in companion animals is not comparable to humans, and most importantly, the cell expansion and activation protocols were not standard, ACT demonstrated notable potential as an innovative treatment for canine malignancies. Our team aimed to evaluate the treatment toxicity of autologous ACT in dogs with OMM because ACT was barely studied in this kind of tumor, and the second endpoint was treatment efficacy. The infusion cells were generated from PBMCs and expanded under the stimulation of IL-15, which was secreted from NK92 feeder cells, as well as IL-2 and IL-21. Our hypothesis is that the infusion would not cause severe systemic toxicity in canine patients while potentially exerting a tumor-control effect.

## 2. Materials and Methods

### 2.1. Patient Enrollment

The study was executed in the National Taiwan University Veterinary Hospital Animal Cancer Treatment Center from July 2021 to December 2022, under the approval of the National Taiwan University Institutional Animal Care and Use Committee (Approval No. NTU-110-EL-00134). Dogs that were recruited in the study had histopathologically diagnosed oral malignant melanoma. Patients were fully staged for metastasis with examinations of their regional lymph nodes and lungs or other distant organs, based on the World Health Organization TNM staging system for dogs with oral melanoma. Stage I: primary tumor size < 2 cm without metastasis; stage II: primary tumor size between 2 and 4 cm without metastasis; stage III: primary tumor size 2–4 cm with metastatic regional lymph nodes, or primary tumor size larger than 4 cm; stage IV was diagnosed if distant metastasis occurred regardless of tumor size or regional lymph node status. The regional lymph node was defined as the mandibular lymph node at the ipsilateral side of the tumor, and any enlarged mandibular or retropharyngeal lymph nodes were surveyed for metastasis through cytology or histopathology. Pulmonary metastasis was checked through 3-view thoracic radiography or computed tomography (CT). Blood examinations were performed before enrollment, as well as during and after treatment, including a complete blood count (CBC) and biochemistry panel. Dogs with severe liver or renal dysfunction or autoimmune disease were excluded. Other clinical staging diagnoses, such as urinalysis or abdominal ultrasonography, were not constrained in every patient but were determined by the clinician. Patients with any clinical stage were allowed to receive the treatment. Receiving concurrent systemic anti-cancer treatments (e.g., chemotherapy, targeted therapy, and other immunotherapies) was not allowed. All the patients’ owners were informed of the study details, and signed consents were obtained.

### 2.2. Autologous Cell Preparation

#### 2.2.1. Generation of Lentiviral-Transduced NK-92/IL-15 Feeder Cell

Canine IL-15 cDNA (NCBI Reference Sequence: NM_001197188.1) was amplified and cloned into the pLAS3w vector. Canine IL-15 lentivirus was generated via transfection of 293T cell line with lentiviral transfer vector (pLAS3w-IL-15), pCMVΔR8.91 plasmid (expression of Gag, Pol, and Rev protein), and pMD.G plasmid (expression of VSV-G protein). All package plasmids (pLAS3w, pCMVΔR8.91, and pMD.G) were obtained from RNAi Core Laboratory in Academia Sinica, Taiwan. NK-92 cells were transduced with the IL-15 lentiviral particles, and cells with high expression were further screened by puromycin (Sigma-Aldrich, Taufkirchen, Germany) and sorted by flow cytometry (Becton-Dickinson, Franklin Lakes, NJ, USA). Transfected NK-92/IL-15 feeder cells were cultured in PRMI-1640 culture medium (Gibco, Grand Island, NY, USA), supplemented with 10% FBS (Invitrogen, Grand Island, NY, USA), 100 IU/mL IL-2 (Proleukin, Clinigen Healthcare, Staffordshire, UK), and 2 mM L-glutamine (Sigma-Aldrich, Taufkirchen, Germany) to maintain gene expression.

#### 2.2.2. Canine PBMC Collection and Infusion Cell Expansion

Autologous blood of 10–20 mL was collected from every tumor-bearing dog. PBMCs were isolated from the autologous blood sample by density gradient centrifugation using Ficoll Plus (eBioscience, Uppsala, Sweden). PBMCs (2 × 10^6^ cells/mL) were incubated with RPMI-1640 medium, supplemented with 10% FBS, 1% Penicillin-Streptomycin (Gibco, Grand Island, NY, USA), 50 μM 2-Mercaptoethanol (Sigma-Aldrich, Taufkirchen, Germany), 2500 IU/mL human IL-2, and NK-92/IL-15 conditional medium (containing approximately 10 IU/mL IL-15) at 37 °C/5% CO_2_ for 14 days. Cells were stimulated with 5 ng/mL IL-21 (R&D Systems, Minneapolis, MN, USA) for the first seven days of culture. The culture medium was replaced with fresh culture medium with IL-2 and IL-15 conditional medium every three days.

#### 2.2.3. Phenotype Analysis of the Infusion Cells

Autologous blood samples from four healthy dog donors (No. 1, four-year-old, male, castrated Standard Poodle; Nos. 2–4, four-year-old, female intact Greyhounds) were collected, and representative infusion cells were obtained under the same culture conditions described above. Phenotype of the representative cell sample was analyzed by Accuri^TM^ C6 flow cytometry (BD, Heidelberg, Germany). The FITC-conjugated mouse anti-dog CD3, PE-conjugated rat anti-dog CD5, and Alex647-conjugated mouse anti-dog CD21 antibodies (all from Bio-Rad, Hercules, CA, USA) were used to stain the representative cell samples. A small cell pellet from donor No.1 was prepared for cytology and stained by the Wright–Giemsa method for cell morphology review.

#### 2.2.4. Cytotoxicity Analysis of the Infusion Cells

In addition to phenotypical analysis, cytotoxicity test of the infusion cells from the healthy donor Nos. 2–4 was performed according to a previously published study [24] and the manufacturing protocol. The cytotoxic effect of the infusion cells was investigated by a CytoTox96^®^ Non-Radioactive Cytotoxicity Assay (Promega, Madison, WI, USA). The M5 canine melanoma cell line (kindly provided by Dr. Michael Kent from UC Davis) was used as the target cell, and the infusion cell was the effector. Briefly, target cells were co-cultured with the infusion cells at various effector/target ratios (E/T) of 10:1, 5:1, and 2.5:1. After 24 h of co-culture, 50 μL of supernatant was used for cytotoxicity measurement. The percentage of cytotoxicity for each E/T ratio was calculated as (experimental culture medium background)−(effector cell spontaneous release−culture medium background)−(target spontaneous release−culture medium background)/(target maximum release−volume correction control−target spontaneous release−culture medium background) × 100.

### 2.3. Autologous Cell Transfusion (ACT) Therapy Protocol

Before use, the cultured infusion cells were washed twice with phosphate-buffered saline (PBS) and the final product was re-suspended in 4 mL 0.9% normal saline and stored in a 10 mL syringe. The final product contained around 5 × 10^7^ cells per dose. The product was restricted to same-day use and was transported from the lab to the hospital under 4 °C. The planning protocol was four doses of weekly administration. For the first-time infusion, blood pressure was measured as a baseline. An intravenous (IV) catheter was indwelled into the patient’s cephalic vein, and diluted diphenhydramine at 1 mg/kg and maropitant at 1 mg/kg were slowly injected before cell infusion. The autologous cell was recovered to room temperature, and the clinician shook the cells to make them evenly distributed by looking to see if fluid became turbid. The ACT was performed via continuous-rate infusions for at least 30 min. After infusion, the patient was required to stay for at least another 30 min for any adverse event monitoring. 

### 2.4. Response and Adverse Event Evaluation

Response evaluation was based on the Veterinary Cooperative Oncology Group Response Evaluation Criteria in Solid Tumors (v1.0) [25]. If the best response was a stable disease, the duration was at least four weeks. A clinical benefit was further defined when the patient achieved stable disease or partial or complete remission. Adverse event evaluation was based on the Veterinary Cooperative Oncology Group—Common Terminology Criteria for Adverse Events (VCOG-CTCAE) v1.1 and v2 [26,27]. Blood examination of an essential CBC and biochemistry panel and 3-view chest radiographs were performed before the first and third treatments and two weeks after the fourth treatment, if no abnormal clinical signs occurred during the whole treatment course. Then, the patients were regularly followed up and re-staged monthly to every three months.

### 2.5. Survival and Statistical Analyses

Several factors that were proven to be prognostic in previous studies, including tumor information (tumor location [1], size [1], bone invasion status [28], and residue tumor before treatment [29]), clinical stage [1,29], histopathological features (mitotic count and lymphovascular invasion) [28,30], and treatments other than ACT were recorded. The response, adverse events of ACT, and peripheral blood neutrophil/lymphocyte ratio (NLR) before and after ACT, were also documented. Toxicity and the progression-free interval (PFI) were the primary endpoints. PFI was defined from the day ACT started to the day tumor progression was recorded. Overall survival time (OST) was also calculated from the day of tumor diagnosis to the day of death for any reason. The survival was described by the Kaplan–Meier curve, and the univariable prognostic factors were analyzed via the log-rank test. A *p* < 0.05 was considered significant. All the analyses were carried out through GraphPad Prism (RRID: SCR_002798), version 9.4.0, GraphPad Software, San Diego, CA, USA, www.graphpad.com.

## 3. Results

### 3.1. Patients’ Characteristics

Ten patients were recruited. The median age was 14 years old (range, 8–16), the median and mean body weights were 7.9 and 11.7 kg (range, 5–24). Four dogs were mixed breed; three were Miniature Poodles; and there was one each of Spitz, Pug, and Shi Tzu. There were seven castrated male dogs and three spayed females. 

All patients had histopathological diagnoses of oral malignant melanomas, and two of them had amelanotic melanoma confirmed by immunohistochemistry stains (Melan-A and/or PNL2). Six patients had their tumor located in the mandible, three in the maxilla, and one in the tongue base. Two of the three maxillary melanomas were rendered non-operable for a wide-margin excision because the tumor invaded the nasal cavity (No. 3) and retrobulbar region (No. 6); patient No. 10 had a hard/soft palate melanoma and received mass-debulking surgery. As for the patients with mandibular tumors, none of them received wide-margin surgery. There were two stage I, two stage II, three stage III, and three stage IV patients. All stage IV patients were diagnosed with a thoracic CT. The regional lymph nodes were confirmed to be metastatic in two patients and reactive in four patients by histopathology, were diagnosed reactive in two patients by cytology, and were not surveyed in two dogs because a stage IV disease was diagnosed. Four dogs had evidence of bony invasion and four did not, while two were not surveyed. Histopathological features like lymphovascular invasion and Ki67 staining were inconsistently recorded, but the mitotic count was regularly calculated in each case with a median number of 13 per 10 high-power fields (range, 4–37).

Three dogs had mass-debulking surgery and maintained microscopic disease before entering the ACT trial. The other seven had macroscopic disease either because of a tumor recurrence or a non-operable status. Eight patients did not receive other systemic treatments before ACT; two switched to ACT after failing other immunotherapeutic trials.

Patients’ characteristics are presented in Table 1.

### 3.2. Phenotype Analysis and Cytotoxicity Test of the Infusion Cells

**Figure 1:**
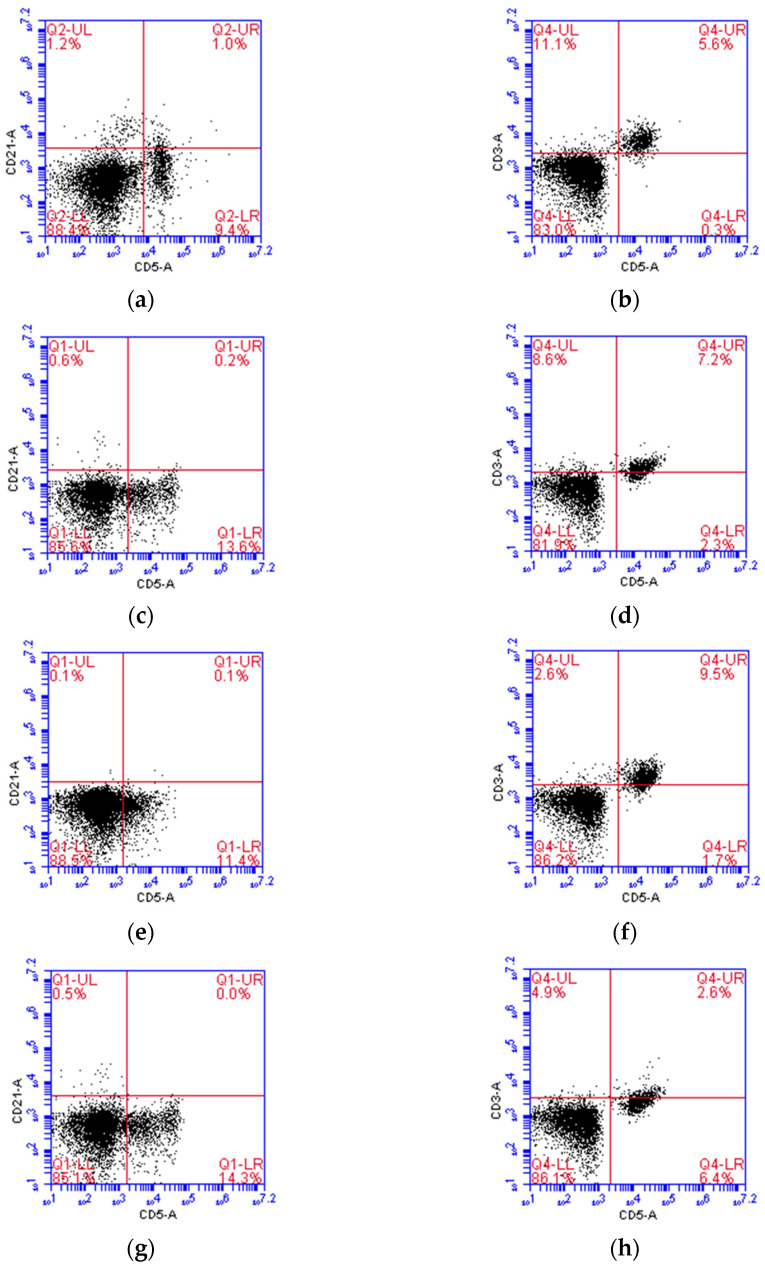
Representative flow cytometric results of the immunophenotype of the in vitro-expanded autologous infusion cells from four healthy donors indicate that the infusion cells were predominantly non-B non-T cells: (**a**,**c**,**e**,**g**) In donor Nos. 1 to 4, the cells were predominantly CD21^−^CD5^−^ (85.1–88.5%), indicating a non-B phenotype; (**b**,**d**,**f**,**h**) In donor Nos. 1 to 4, the cells were predominantly CD3^−^CD5^−^ (81.9–86.2%), indicating a non-T phenotype.

**Figure 2:**
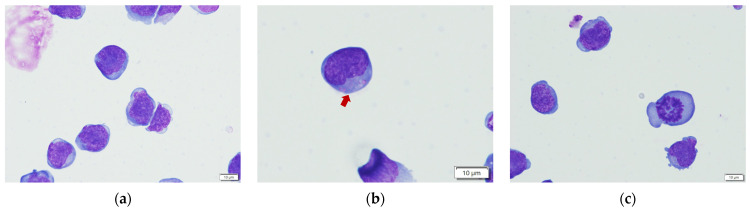
Representative morphological results of the infusion cells. (**a**) The medium to large lymphocyte with high N/C ratio; (**b**) The intracytoplasmic granules (red arrow) were occasionally seen, representing a possible feature of NK cells; (**c**) The mitotic figure.

In canine NK cell studies, CD5^dim^ was thought to be one of the NK cell markers, and CD5^−^CD3^−^CD21^−^ was a presumptive phenotype of NK cell at some degree of maturation [31]. Our flow cytometric results of the representative infusion cells indicate that the predominant phenotype was non-B/non-T cells and was uniform among the four healthy donors, as 85.1–88.5% of the cells were CD5^−^CD21^−^ and 81.9–86.2% were CD3^−^CD5^−^. Morphologically, the representative cells were almost medium to large round cells, with a high nucleus/cytoplasm ratio and occasionally intracytoplasmic azurophilic granules, representing NK cell features. Some mitotic figures also existed, indicating cell division ability. The results of flow cytometry and cell morphology are presented in Figure 1 and Figure 2.

**Figure 3:**
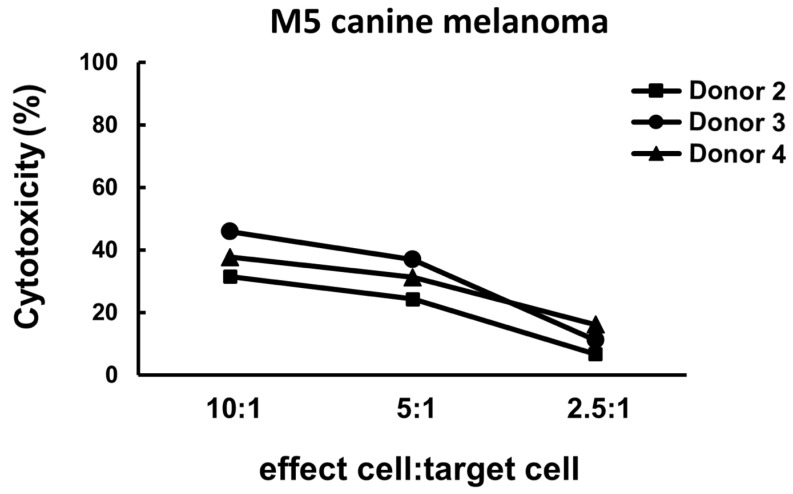
Representative cytotoxicity results of the infusion cell samples obtained from three healthy dog donors. The cytotoxicity was around 30% to nearly 50% when the E/T ratio was 10:1, was mildly decreased when the E/T ratio was 5:1, and dropped to less than 20% when the E/T ratio decreased to 2.5:1.

The result of cytotoxicity Is shown In Figure 3. The infusion cells exhibited cytotoxicity against the M5 melanoma cell line. The effect was highest when the E/T ratio was 10:1 (around 30% to nearly 50%) and diminished when the E/T ratio decreased.

### 3.3. Adoptive Cell Therapy and Adverse Events

All ten patients strictly followed the infusion protocol. Six patients finished the planned four infusion doses, and two of them received two more infusions (extended into a total of six doses) as the owners requested. One patient received only three doses because of the constant and rapid tumor growth. Generally, the treatment was well tolerated in the seven dogs. Three patients did not experience any side effects throughout their treatment course; three dogs had a grade 1 appetite alteration with no need for medical intervention; while one patient developed a grade 1 elevated ALT after two infusions, and the condition recovered with symptomatic liver-protective medications. The owner of patient No. 7 reported a suspected fever after the third infusion but no diagnosis was performed, and the condition recovered without any medical intervention. No similar episode was observed after the fourth infusion.

However, the other three patients had suspected infusion-related anaphylaxis. Although no treatment-related death occurred, this still increased the safety concern. One patient (No. 9) developed facial swelling and itching around two hours after the first infusion and recovered after steroids and anti-histamine administration. The clinical signs were likely to be a hypersensitivity reaction, so the patient was withdrawn from the trial. Two patients (No. 8 and No. 10) received their first three infusions without scathe, but during the last treatment, suspected anaphylaxis episodes occurred after around 3 mL of cell fluid infusion. Patient No. 8 developed excessive panting, ptyalism, and mild cyanosis. The infusion was terminated, and oxygen was provided immediately; another dose of 1 mg/kg of diphenhydramine was administered. The patient recovered soon with normal vital signs, blood pressure, SpO_2_, and heart rate. Patient No. 10 developed severe bradycardia, hypotension, unconsciousness, and hypersalivation during the fourth treatment, also after infusing 3 mL of cell fluid. An anaphylactic shock was highly suspected. Diphenhydramine, dexamethasone, epinephrine, and fluid therapy were administered. The patient then recovered and was hospitalized overnight for further monitoring. Because no blood exam was required before the fourth infusion, a follow-up blood test was performed the next day. The CBC revealed mild leukocytosis (WBC 20,000/μL), and the biochemistry was generally normal. The patient was discharged peacefully, and the owner chose to take active surveillance of the tumor. The treatment information of each patient is presented in Table 2.

### 3.4. Outcome

Patient No. 9 was excluded from the data analysis because only one dose of ACT was administered. Patient No. 1 was censored from the PFI analysis because no tumor progression was observed at death. All the other eight dogs had confirmed or suspected tumor progression before death. During the treatment course, three dogs had progressive disease and six had stable disease or were in progression-free status and the clinical benefit rate was 67%. Patient No. 10 presented with a generalized seizure 170 days after ACT. An intracranial mass was detected on the brain MRI, which could not be determined as a melanoma metastasis or a primary brain tumor. The owner sought RT through some private access, and a hypofractionated radiation therapy was performed against the solitary brain tumor. However, on the post-RT follow-up CT, several intracranial and pulmonary masses were identified, and melanoma metastasis was suspected. The owners decided on euthanasia due to deteriorated neurological signs; the survival was 279 days. No necropsy was performed. 

Seven patients expired for confirmed or suspected tumor-related reasons. Patient No. 1 died because of cardiac disease without evidence of tumor progression. Patient No. 6 developed a pulmonary tumor 84 days after ACT and was euthanized three months later because of pleural effusion and respiratory distress. A primary epithelial tumor was highly suspected according to the computed tomography and fine-needle aspiration results, and the oral melanoma exhibited stable to mild progression during the whole course. 

**Figure 4:**
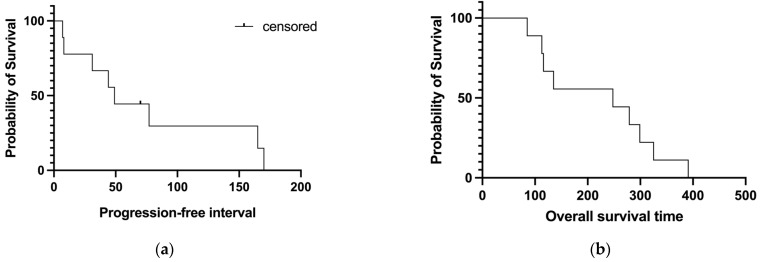
Median progression-free interval ((**a**); 49 days) and overall survival ((**b**); 248 days) of the patients enrolled.

Although it was not the primary study aim, the median PFI of this group of dogs was 49 days (Figure 4a) and the median OST was 248 days (Figure 4b).

Several factors were analyzed univariably for prognostic significance (Table S1). After subdividing the patients into the progression disease group (PD) and the achieving clinical benefit group (non-PD), a significantly shorter PFI was found in the PD group (PFI: 26 days vs. 77 days in the non-PD group; *p* = 0.022). For overall survival analysis, receiving other treatments before ACT (*p* = 0.03) was significantly related to prolonged survival. In addition, it was unexpected that patients in the PD group (*n* = 3) also had a significantly longer OST (*p* = 0.01). Regarding the three dogs with PD, two of them (patient No. 6 and No. 7) had previous anti-tumor treatment (a dendritic cell-based immunotherapy constructed by our institution) before entering the ACT trial, and patient No. 7 also received chemotherapy after failing ACT. The survival rates of these two dogs were 299 and 391 days, resulting in a longer median OST of the PD group because of the small population. Because of the small sample size, no multivariable analysis was performed.

The outcome of the ten patients is summarized in Table 2.

## 4. Discussion

This manuscript reported a pilot study of ACT application in canine OMM, and to the author’s knowledge, this is the first clinical trial of ACT in this tumor. In practical terms, dogs with OMM in Taiwan face limited treatment options except for surgery and chemotherapy. Radiation therapy has been unavailable since 2020, and we do not have the DNA vaccine Oncept^TM^. Searching for novel treatment modalities and establishing appropriate clinical trials are essential for dogs with OMM. Consequently, we conducted this pilot study and applied the ACT in dogs with non-operable oral melanomas or whose owners declined other treatments. The preliminary results of the ACT indicate that the treatment was generally tolerable, adverse events were predominantly gastrointestinal discomforts, and no treatment-related death was reported, but the possible anaphylaxis reaction should be a concern. The median PFI of the ACT was 49 days, and the median OST was 248 days. Although the different treatments could not be compared directly due to varying inclusion criteria and patient characteristics, previous research on adjuvant chemotherapy has reported a PFI of eight months [32], and the multimodal treatments incorporating Oncept^TM^ revealed an OST of 510 days [33]. In our institution, a similar group of OMM, consisting of 31% stage I/II and 69% stage III/IV dogs, who received chemotherapy alone, exhibited a median PFI of 42 days and an OST of 181 days (manuscript in press), which did not statistically differ from the current ACT survival.

The infusion cell culture protocol in our study had some differences compared to previously published protocols. Briefly, we conducted a feeder NK cell that consistently expressed IL-15, a vital factor in simulating NK cell differentiation [31,34]. Because IL-2 is the main cytokine used in human LAK and TIL cell activation and expansion [10,11,35], we also added IL-2 and IL-21 to our culture process, similar to the protocol employed by O’Connor’s group [14]. Our phenotype analysis of the final cell product revealed predominantly non-B non-T CD5^−^CD3^−^CD21^−^ cells; microscopically, the cells occasionally contained intracytoplasmic granules. The results are uniform among different healthy dog donors. We were aiming to expand NK cells, but we did not have other access for further determination. However, referring to the study of canine NK cell isolation and characterization [36], we presumed that our infusion cells primarily comprised NK cells, which was supported by the evidence of the immunophenotype and the in vitro cytotoxicity against the canine M5 melanoma cell line. There was no CD3 depletion procedure, so it could be expected that a small percentage of T cells existed. In human research, early clinical studies of the LAK therapy generated the natural killer cells by culturing and stimulating the PBMCs with IL-2, while a more recent CIK cell treatment used an anti-CD3 antibody, IL-2 and IFN-gamma, to obtain a heterogenous cytotoxic T-cell population. Given our culture condition, immunophenotype, and morphology and the cytotoxicity analysis of the infusion cells, we considered our ACT to align more closely with the LAK cell therapy. The variations in manufacturing methods among standard LAKs in human research, previous ACT in the veterinary field, and our ACT in the current study may partly explain the varied clinical outcomes. Furthermore, alternative explanations of the anti-tumor effect of the infusion cells are worth considering. In the current study, we performed a cytotoxicity test by using expanded infusion cells from healthy donors, and the result indicates that the infusion cells exhibited cytotoxicity against the M5 cell line. However, clinically, we utilized autologous PBMCs from tumor-bearing dogs for treatment. It has been reported that the expansion and cytotoxicity of PBMC-derived NK cells following IL-2 stimulation were deficient in dogs with neoplasms compared to healthy dogs [37]. Therefore, it may also be plausible that the intrinsic NK cell insufficiency in tumor-bearing dogs resulted in a lower proliferation ability and cytotoxicity of the expanded infusion cells, leading to inadequate antineoplastic activity. In addition, because we performed the cytotoxicity analysis by using a cell line, whether the infusion cells from individual patients were cytotoxic specifically, or whether the in vitro cytotoxicity could directly represent in vivo anti-tumor efficacy and correlate with clinical outcome, is uncertain currently. Lastly, the dosage of our ACT was around 5 × 10^7^ cells per dose, which was not calculated based on body weight or body surface area. The dosage differed from previous canine studies, and the total cell count was lower. In human CAR T-cell therapy, increasing cell dosage led to a higher response and toxicity until a threshold was reached [38]. However, in the current study, we did not determine the cell dosage threshold for optimal efficacy.

The primary objective of the current study was to assess the safety of our ACT in OMM dogs. The initial seven dogs enrolled experienced no or only mild toxicities, with a grade 1 hyporexia being the most common adverse event. However, the subsequent three dogs presented with suspected hypersensitivity reactions of varying degrees despite receiving diphenhydramine pretreatment. Among them, patient No. 9 developed clinical signs of facial swelling and itching approximately two hours after the first infusion, indicating acute hypersensitivity. Patient Nos. 8 and 10 exhibited anaphylactic reactions during the fourth infusion, characterized by panting, ptyalism, bradycardia, loss of consciousness, and hypotension. Side effects reported in prior ACT studies in the veterinary field were generally not severe and mostly were gastrointestinal disturbances [14,16]. In the recent multimodal immunotherapeutic clinical trial of canine osteosarcoma, one dog received activated T cells without premedication and developed fever, lethargy, vomiting, and erythema immediately after infusion [16]. Although cytokine level was not measured in this dog, the clinical signs were consistent with cytokine release syndrome (CRS). After standardizing pretreatment with diphenhydramine (2 mg/kg IM), maropitant (1 mg/kg SC), and firocoxib (5 mg/kg q24h) in the following twelve dogs, along with washing infusion cells three times instead of once at harvesting, no similar acute reaction happened again. Another case report of autologous CAR T-cell infusion in a dog with B-cell lymphoma documented a CRS after treatment, as evidenced by several subsequently increased serum cytokine levels [9]. In the current study, although a cytokine analysis was not performed in patient No. 8 and No. 10, a cytokine storm was still a possible differential as inappropriate immune responses might be triggered in the two patients. It has been reported that in vitro-expanded CIK cells could secrete cytokines [39], and exosomes might also be released by the cells during the culture process. After repeated infusions, patients could potentially develop a direct cytokine-induced or secondary immune-responsive hypersensitivity against these components. On the other hand, because we did not perform a T-cell selection or depletion, a small percentage of T cells may exist and initiate immune reactions. In human research, an iatrogenic cytokine storm can be induced by CAR T-cell therapy, as well as other T-cell-engaged immunotherapies, gene therapies, immune checkpoint blockades, and allogenic stem cell transfusions. A cytokine storm is characterized by elevated circulating cytokine levels, acute systemic inflammatory syndrome, and secondary or cytokine-driven organ failure [40]. Fever is also a clinical hallmark of CRS. Although none of our patients had fever after treatment, and neither circulating cytokines were evaluated nor evidence of systemic inflammation existed, we still cannot rule out an ACT-induced cytokine storm. Bacterial contamination was less likely to be the reason because no abnormality was observed during the culture, but endotoxin could not be completely ruled out because no examination was performed. Processing cell products under good laboratory practice guidelines is mandatory for future trials.

Regarding the outcome analysis, the prognostic correlation should be explained with caution because the dogs recruited in this study had heterogeneous characteristics. The authors hope to report as much information as possible based on this population. Disease progression during the ACT course was a significant negative prognostic factor for median PFI in the current study, which was reasonable. However, patients with progressive disease during ACT had significantly longer overall survival, and this was not expected but explainable. Two out of three patients in the PD group received other treatments before receiving ACT. Because we calculated the overall survival from the day of diagnosis to death, the longer survival times of these two dogs can be related to tumor suppression induced by other treatments, and the overall survival would be affected due to the small sample size. In addition, both the two dogs received dendritic cell-based immunotherapy developed by our team (manuscript in preparation) before entering the ACT trial, and one had chemotherapy after ACT failure; there might be a synergistic effect of ACT and dendritic cell-based immunotherapy or chemotherapy, leading to a slower tumor progression. Further investigation is needed to answer this question. Another prognostic factor mentioned by Noguchi et al. was bone invasion, which might affect the RT outcome through hypoxia [41]. In the current study, the population of bone involvement and PD group overlapped, and because we did not perform a multivariable analysis, the effect of bone invasion on survival could not be truly determined, and the correlation of bone lysis and dogs with OMM receiving immunotherapy could not be further addressed. Other prognostic factors reported in canine OMM did not exhibit significance in the current study, and the most reasonable explanation was the enrolled patients’ heterogeneity. 

The current study has several limitations. Firstly, the sample size was small, and certain features, such as clinical stage and residue tumor status, were not evenly distributed. Although we compared a similar group of patients in our department who had chemotherapy as their sole systemic treatment, we did not enroll a non-treated control group. This limitation would raise the question of whether the outcomes of patients receiving ACT would differ if they had less-advanced stages or had microscopic tumors before treatment, as immunotherapy is thought to be more potent in an adjuvant setting. It was also plausible that the heterogeneous patient characteristics of this small group made it difficult to generate a proper conclusion about treatment response and outcome. We hypothesized that the cell therapy could be more effective in dogs with earlier clinical stages and microscopic disease. Therefore, after treatment protocol modification, we plan to enroll a more significant and specified group of dogs with stage I/II OMM receiving adjuvant cell therapy to further clarify the treatment efficacy. Regarding our in vitro-expanded cells, we only identified the phenotype as non-B non-T lymphocytes. CD5^−^CD3^−^CD21^−^ was thought to be a feature of canine NK cells at some degree of maturation, and CD5^dim^ was also a potential phenotypic marker, especially when culturing with IL-2 [31]. Our results are consistent with this feature, but we could not further determine, for example, the expression of another canine NK cell marker NKp46 [21,31]. Specific NK cell markers, such as CD16 and CD107a, representing NK cell activation and degranulation, were found in human research but have not been clearly determined in the veterinary field [31,42]. Because we lacked canine-specific antibodies, we did not perform these marker expression analyses. We also did not evaluate the peripheral blood lymphocyte composition and serum cytokine changes before and after treatment. Although it may not directly correlate with actual in vivo efficacy, it could still be considered in the future to investigate what influence the ACT could elicit. Furthermore, we did not track the infused cells, preventing us from determining whether these cells homed to the local tumor site or persisted in killing circulating tumor cells in the peripheral blood or lymph nodes. In addition, in the patients with suspected anaphylaxis, we did not perform a serum cytokine analysis to find out whether there was a cytokine-releasing syndrome. 

Despite these limitations, our interest in further exploration of the ACT in canine malignant neoplasms remains undiminished. For protocol modification, the infusion cells could be washed more times during harvesting, and premedication of intramuscular diphenhydramine of 2 mg/kg and firocoxib, referring to Flesner et al. [16], could be utilized to prevent a possible cytokine storm. Regarding cell dose and treatment efficacy, a body weight- or body surface area-based cell count calculation, as in previous studies, is reasonable. On the other hand, a dose-escalation trial at a lower cell dose could also be considered to help optimize treatment efficacy without causing severe adverse events. Most importantly, a good manufacturing practice-based culture protocol should be mandatory in the future. In human research, cell therapy can be combined with other conventional anti-tumor therapies or immunotherapies. For instance, it has been reported that combination treatment yielded efficacy against radiation- or chemotherapy-resistant tumor cells, or enhanced the tumor-control effect of chemotherapy [43,44,45,46]. The recent adoptive T-cell transfer in the canine osteosarcoma trial also combined ACT with a tumor vaccine and IL-2 injection [16]. This evidence illustrated the potential for further research in canine cancer management and mechanisms of cell therapy in human cancers, as dogs are well-accepted animal models.

## 5. Conclusions

The current study reported a pilot clinical trial of an autologous adoptive cell transfer in ten dogs with oral malignant melanoma. The ACT provided limited tumor control effects, and suspected treatment-related anaphylaxis was observed in three patients. Given that most patients in the current study had advanced clinical stages and macroscopic disease, we deem it necessary to conduct a further trial in patients with less advanced disease. We suggest establishing a standardized cell therapy protocol in dogs, with intensive monitoring of blood biomarkers during treatment. Multimodal treatment of ACT and other conventional therapies or immunotherapies in canine OMM is also worth further evaluation.

## Figures and Tables

**Table 1:** Descriptive information of the ten patients’ characteristics in the ACT trial.

Patient No.	Breed	Age (y/o)	Sex	BW (kg)	Tumor Diagnosis	Oral Mass Location	Tumor Size ^1^	Clinical Stage	Regional LN	Mitotic Count/10HPF	Bone Invasion
1	Spitz	15	FS	6.7	AMM	Lt. mandible	1.6 cm	I	Non-meta	24	No
2	Mixed	8	MC	24	MM	Tongue	6 cm	IV	Unknown	18	No
3	Mixed	13	MC	21	MM	Rt. maxilla w/nasal cavity	4.8 cm	IV	Unknown	5	Yes
4	Miniature poodle	16	MC	5	MM	Lt. mandible	3 cm	III	Metastatic	4	Unknown
5	Pug	14	MC	8	MM	Rt. mandible	4.3 cm	III	Reactive	6	Yes
6	Miniature poodle	13	FS	5.8	MM	Lt. caudal maxilla	2 cm	III	Reactive	8	Yes
7	Shi Tzu	14	MC	5.5	MM	Lt. mandible	1 cm	IV	Metastatic	37	Yes
8	Miniature poodle	12	MC	7.8	MM	Lt. mandible	1 cm	I	Non-meta	19	Unknown
9	Mixed	14	FS	15	AMM	Lt. mandible	4 cm	III	Reactive	4	Unknown
10	Mixed	14	MC	18	MM	Maxilla, hard/soft palate	2.5 cm	II	Non-meta	23	No

^1^ The tumor size was the maximum diameter of the patient’s oral mass size before entering ACT, except for patient No. 7, who received ACT without local recurrence but a solitary macroscopic pulmonary metastatic lesion, measured around 1 cm on radiographs. Abbreviations: FS, female spayed; MC, male castrated; AMM, amelanotic malignant melanoma; MM, malignant melanoma.

**Table 2:** Descriptive information of ten patients’ treatment and outcome in the ACT trial.

Patient No.	Surgery of Oral Mass	Other Therapies before ACT	Residue Tumor	ACT Doses	Best Response	Side Effects	PFI (Days) and Progression Site	OST (Days) and Cause of Death
1	Marginal excision	No	Microscopic	4	PF	No	70 days from ACT to death	116; Cardiac disease
2	Tongue mass debulking	No	Macroscopic/Lung	6	SD ^1^	Grade 2 elevated ALT	31; pulmonary	85; Lung metastasis
3	Biopsy	No	Macroscopic/Oral and lung	6	SD	No	44; pulmonary	135; Primary tumor and lung metastasis
4	Biopsy	No	Macroscopic	4	SD	Grade 1 hyporexia	165; oral	248; Pulmonary epithelial tumor
5	Debulking	No	Macroscopic ^2^	3	PD	No	7; oral	113; Primary oral tumor
6	Partial debulking ^3^	Yes	Macroscopic	4	PD	Grade 1 hyporexia	8; oral	299; Primary oral tumor
7	Debulking	Yes	Macroscopic/Lung	4	PD	Grade 1 hyporexia, susp. fever after 3rd dose?	49; pulmonary	391; Lung metastasis
8	Marginal excision	No	Macroscopic ^2^	4	SD	Susp. hypersensitivity, 4th dose	77; oral	325; intra-RA mass, not sure if tumor related
9	Debulking	No	Microscopic	1	NA	Grade 1 hypersensitivity, 1st dose	NA	272; Primary oral tumor
10	Debulking	No	Microscopic	4	PF	Susp. anaphylactic shock, 4th dose	170; intracranial	279; Multiple intracranial and lung masses

^1^ The owner of patient No. 2 declined a follow-up X-ray during treatments; the stable disease was determined mainly based on clinical signs. ^2^ Patients had oral tumor-debulking surgery, but tumor recurred when they received ACT. ^3^ Partial debulking: patient No. 6 had tumor predominantly in the oral cavity, but the tumor invaded the orbital bone and retrobulbar region, which could not be removed by surgery, thus the surgeon only excised the mass in the oral cavity as much as possible. Abbreviations: PF, progression free; SD, stable disease; PD, progression disease; RA, right atrium; NA, not assessed, because patient No. 9 was withdrawn from the treatment.

## Data Availability

The data presented in this study are available in the article.

## Supplementary Materials

The following supporting information can be downloaded at: https://www.mdpi.com/article/10.3390/vetsci11040150/s1, Table S1: Univariable analysis of prognostic factors of the ten patients in the ACT trial.
