# Supplementary material for: Limited Clinical Efficacy with Potential Adverse Events in a Pilot Study of Autologous Adoptive Cell Therapy in Canine Oral Malignant Melanoma
Source: Vet Sci. 2024 Mar 28;11(4):150. doi: 10.3390/vetsci11040150 (PMC11053650; doi:10.3390/vetsci11040150)
Supplement: Supplementary file 1 [file vetsci-11-00150-s001.zip › vetsci-2916650-supplementary.pdf]

veterinary sciences

MDPI

Table S1. Univariable analysis of prognostic factors of the ten patients in the ACT trial

**Analyzed Factors**	**n**	**PFI (days)**	***p* Value**	**n**	**OST (days)**	***p* Value**
Other therapies before ACT			0.25			0.03 *
No (primary tumor)	6	77		7	135	
Yes (recurred tumor)	2	28.5		2	358	
Location			0.88			0.43
Maxilla	3	44		3	279	
Mandible	4	77		5	248	
Tumor size			0.87			0.33
>=median 2.5 cm	5	44		5	248	
< median 2.5 cm	3	63		4	220	
Bone involvement			0.12			0.03 *
Yes	4	26		4	312	
No	3	77		5	116	
Regional lymph node			0.89			0.93
Non-metastatic	4	77		5	286.5	
Metastatic	2	107		2	279	
Tumor stage			0.06			0.19
Early (stage I/II)	2	123.5		3	116	
Late (stage III/IV)	6	37.5		6	273.5	
Residue disease			0.058			0.54
Macroscopic	7	44		7	248	
Microscopic	1	170		2	197.5	
Mitotic count			0.22			0.3
>=median 13/10 HPF	4	77		5	116	
<median 13/10 HPF	4	26		4	273.5	
NLR change			0.43			0.83
Increase	5	44		5	248	
Decrease	3	49		3	279	
Treatment response			0.036 *			0.01 *
PD	3	8		3	325	
Non-PD	5	77		6	125.5	
Adverse events			0.55			0.54
Yes	6	63		6	263.5	
No	2	44		3	135	

* The survivals were significantly different. Abbreviations: NLR, neutrophil-to-lymphocyte ratio
